# The social self: integration of multimodal communication signals in dyadic interactions

**DOI:** 10.1098/rstb.2025.0164

**Published:** 2026-11-10

**Authors:** Kai Vogeley, Mark Wünsche, Mathis Jording

**Affiliations:** ^1^ Department of Psychiatry, University Hospital Cologne, Cologne, North Rhine-Westphalia, Germany; ^2^ Institute of Neuroscience and Medicine – Cognitive Neuroscience INM3, Research Center Juelich, Juelich, Germany

**Keywords:** multimodal communication, chronemics, autism spectrum disorder, individual self, social self, double empathy theory

## Abstract

Humans are in continuous exchange with other persons, thereby integrating the individual self and the social self. This requires the production of different verbal and nonverbal signals at the subpersonal level (individual self) and, at the same time, their coordination with various perceived signals at the interpersonal level (social self). Many of our communicative signals are conveyed substantially nonverbally—that is automatically, intuitively and effortlessly, requiring dynamic coordination over time (‘chronemics’). Whereas successful communication is usually perceived as pleasant and satisfying, communication can also be experienced as disrupted if chronemics do not unfold properly. With respect to the particularly informative example of autism spectrum disorder, we propose the concept of ‘double invisibility’. Non-autistic persons intuitively produce and expect a rich, multimodal stream of communication signals without being aware of it (first invisibility). By contrast, autistic persons produce and expect nonverbal cues either to a significantly lesser extent or not at all without being aware of it (second invisibility). This mismatch leads to misunderstandings and disturbed communication. The coordination within the dyad that is continuously constructed through real-time communication is crucial to understand the full complexity of multimodal interactions and their disturbances.

This article is part of the discussion meeting issue ‘From being to identity: analysis and synthesis of the self’.

## 1. The individual self and the social self

In our everyday life, we are constantly shifting between self-reflective, introspective or contemplative states, in which we reflect on ourselves and our own inner states, and interactions and communication with others, during which we must also take into account the inner states of others. Technically speaking, one can distinguish between the ‘self as subject’ or individual self—as I experience myself—and the ‘self as object’ or social self—the self as perceived by others. This very basic distinction goes back to the earliest accounts of modern psychology; already James distinguished between ‘I’ and ‘Me’ [1,2]. In the conceptualization of Neisser, the interpersonal self is one facet besides the private self; it relates to the ‘individual engaged in social interaction with another person’ [3, p. 18]. In the philosophical tradition, Mead has proposed a close connection between the individual development and the formation of the community to which the individual belongs. To distinguish between them, Mead uses again the two terms ‘I’ and ‘Me’. While the ‘I’, from the perspective of the first person, indeed denotes a phenomenal experience of ourselves, the ‘Me’ refers to a reflection of what the social environment conveys to us about ourselves. Both instances are in close interaction with one another. The ‘Me’ is the sum of attitudes of others towards oneself; the ‘I’, in contrast, is the response of the individual to the attitudes of the others [4].

The related cognitive capacities can be described as self-other-differentiation and self-other-exchange. Only the clear distinction between one's own and others’ mental states allows one to ascribe mental states to others in the process of self-other-exchange [5]. Both capacities mutually require each other. A more detailed view was provided by cultural psychology, differentiating an independent from an interdependent self. Whereas the independent self stresses the need to maintain the individual's independence from others by attending to oneself and by developing and expressing one's own attributes, the interdependent self, in contrast, stresses the fundamental relatedness of individuals to others, emphasizing attending to others and being in harmonious interaction with others. The independent self refers to individualism and individualistic cultures, namely groups of individuals who experience themselves as having a relatively separate identity against others; in contrast, the interdependent self refers to collectivism, namely groups of individuals whose identity is strongly defined by long-lasting group memberships [5,6].

## 2. Communication

To successfully navigate the social world, evolution has equipped humans with different communicative abilities, to adjust to social affordances and to adapt and coordinate intentions, feelings and actions with others or, in short, with a ‘we’-mode [7]. ‘We can never abstain from communication, and as human beings and members of a society, we are biologically compelled to communicate’ [8, p 6]. Even if signals are not intended to convey any concrete meaningful message, they are interpreted as if others want to tell us something: ‘all actions and events have communicative aspects, as soon as they are perceived by a human being’ [8, p. 6]. In the terminology of Watzlawick, the fact that ‘one cannot not communicate’ describes the first out of five axioms of communication. Besides content, communicative encounters always comprise a very strong relational component. The relation shapes the communicative encounter between two partners so that the exchange of content becomes possible. This is the second axiom of communication [9, p. 54].

Communication, in general, thereby coordinates interaction, including discourse and dialogue, manages attention and establishes common ground [10,11] as preconditions for self-construal, social bonding, cooperativity and the development of sciences, technology and arts [5,6,12]. With respect to content, communication is based on the exchange of information or knowledge conveyed by meaningful communicative messages. According to symbolic interactionism, introduced by Mead's distinction of the ‘I’ and the ‘Me’ [4], (i) we (inter)act on the basis of interpretable meanings, (ii) meanings develop during the interaction between persons, and (iii) meanings can change over time [4,13,14]. In other words, rather than being a fixed, pre-given property of an isolated individual self, the social self can be understood as continuously constructed, negotiated and expressed through this real-time, multimodal exchange with others [4,6].

Besides language as communication through words and symbols, research during the last decades has provided convincing evidence that social understanding, the experience of rapport and successful collaboration substantially depend on nonverbal and paraverbal (intonation) communication [15–18], far beyond interpersonal coordination in collaborative motor tasks [19,20]. Nonverbal cues, including, for instance, gaze behaviour, facial expression, body postures and gestures supporting interpersonal coordination, are the ‘social glue’ in human interactions and groups [21]. In particular, nonverbal communication serves different functions, including (i) modelling and coordination functions, (ii) discourse functions, (iii) dialogue functions, and (iv) socio-emotional functions [11]. With respect to the outcome or success of our communication, nonverbal behaviour has a fundamental influence as it shapes the relationship between both partners [15,16]. Surprisingly, we can understand ‘the complexity of feelings and actions’ of another person ‘at a glance’ although ‘the full significance of man's relation to man may not be directly evident’ [22], ‘even the direction of a glance may provide a strong hint as to what the person is thinking, feeling, and wishing’ [22, p. 2]. This canonical work of Heider laid the foundation for the later proposal of the so-called attribution theory in social psychology [23] which integrates actual stimuli (e.g. a smile), the actual situational context and previous knowledge about the communication partner to generate a person's perception judgement about the other person with whom one communicates.

Communication is what helps us to understand other persons in their future-directed goals and intentions and to successfully interact and cooperate with them, essentially based on our mentalizing capacities [24,25]. Communication is required because persons are fundamentally different from physical things by their inherent ambiguity and uncertainty [22]. Persons have inner experiences that let them develop intentions-to-act and goal-directed behaviour so that they become ‘action centers’ [22]. Because of this freedom to make (latent) choices for one's own behaviour, persons are inherently unpredictable; hence, the capacity to exchange and communicate with others is essential [25]. Speculatively, the necessity to communicate with others might be the reason for the early ontogenetic onset of related social abilities. For instance, the phenomenon of joint attention that allows a young child at the age of nine months to manipulate the attention of their carer is a convincing example of an early development of a substantial communication mechanism [26,27]. It has been summarized as the so-called dual function of gaze [28]: gaze not only allows one to perceive the partner but also signals attention and interest for the partner within the shared environment. Subsequently, this capacity can establish a triadic interaction, namely a three-way relationship between *indiviual* self, the other and the object world [29]. A special case is ‘joint attention’ with gaze-contingent agents that is experienced as interactive [27,30–35].

## 3. ‘Chronemics’ of communication

Temporal coordination in social interaction has been studied across several partly overlapping traditions, including substantial developmental work on carer–child interaction [36,37], referred to as ‘interpersonal coordination’ [38], ‘interactional synchrony’ [39] or ‘social entrainment’ [40]. We suggest using interpersonal coordination as an umbrella term that also covers more specific forms of coordination such as synchrony and entrainment. What remains insufficiently integrated across these research fields is that communication is a dynamic, interactive and multimodal process that exhibits temporal interdependencies across both interpersonal and intrapersonal levels, reflected in time-sensitive contingency patterns as socially emergent phenomena (figure 1).

**Figure 1.**
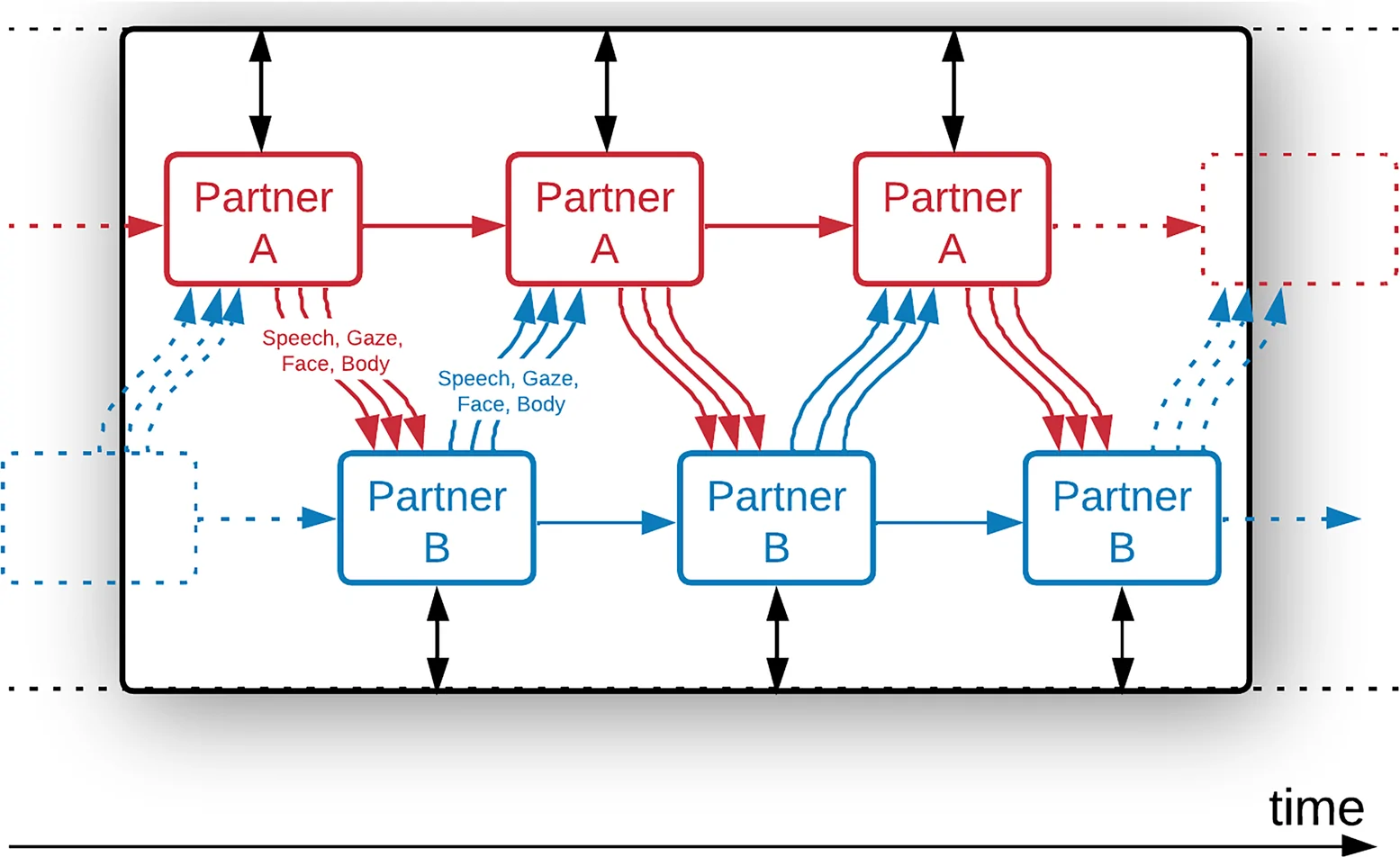
Multimodal stream of communication. Schematic illustration of the multimodal stream of communication signals between two interlocutors. Signals are sent out by one partner and processed by the other who, in turn, responds, thus constituting a ‘social situation’ unfolding over time [8]. Both partners thereby shape their communicative experience, also referred to as rapport (black lines and arrows).

While persons communicate with each other verbally, paraverbally (intonation and prosody) and nonverbally (gaze, gestures and facial expression), they exert an influence on their partner which, in turn, might change the way how the other person thinks, feels or communicates. This creates a dynamic process out of which different patterns of communication can emerge, for instance of a lively discussion with many overlaps in the speech behaviour of both partners or, alternatively, of a boring monologue with only one person speaking over a longer period. At the same time, contingencies in the exchange of communicative cues shaping the social self of both interlocutors are also perceived by the interactants and shape the rapport experience of how well the partners come along with each other [18].

This global experience of the success of the ongoing communication, in turn, mutually modifies the internal plans of both partners. For instance, research on turn transitions during ongoing conversation suggests that the timing of eye gaze directed at the partner plays an important role during the change of the speaker's role [41,42]. Here again the dual function of gaze [28] becomes apparent, that is the fact that gaze does not only allow the speaker to observe their interlocutor but also to signal their intentions and to coordinate the conversation by deliberately averting their gaze or looking at the interlocutor. Concretely, a systematic review showed that directed gaze of the speaker towards the listener before turn-taking is one of the most reliable predictors of turn transitions [43]. Such findings show convincingly that the dyad is the fundamental unit of analysis in the study of the ‘dynamic, ongoing, ever-changing, continuous’ process of communication that does not have ‘a beginning, an end or a fixed sequence of events’ [44, p. 2]. The classical serial sender–receiver concept [45] has to be extended by a systemic approach that takes these emergent contingency patterns of communicating dyads into account—in contrast to the isolated behaviour of detached individuals.

Key to understanding and exploring these contingency patterns is the temporal organization of communication, also referred to as ‘chronemics’ [46]. Timing has been proposed to play a key role in communication already decades ago [47,48]. Chronemics, as a general term, refers to the study of various concepts and processes of human temporality covering objective time (relation to physical clock time) and subjective time (structure of time and time passing by) or any time-related aspects of human communication and interactions. It can be related to different components of the structure of time, including the past (associated with the concept of causality), the present and the future (associated with the concept of finality or goal-directedness). Different modalities operate on different time scales (e.g. gaze: milliseconds; body posture: seconds). The broad definition of the term interpersonal coordination encompasses all forms of alignment between two or more interaction partners in a shared environment, irrespective of whether interactants align deliberately and/or consciously, whether they act simultaneously or shifted in phase and whether actions are mirrored or complementary (figure 2).

**Figure 2.**
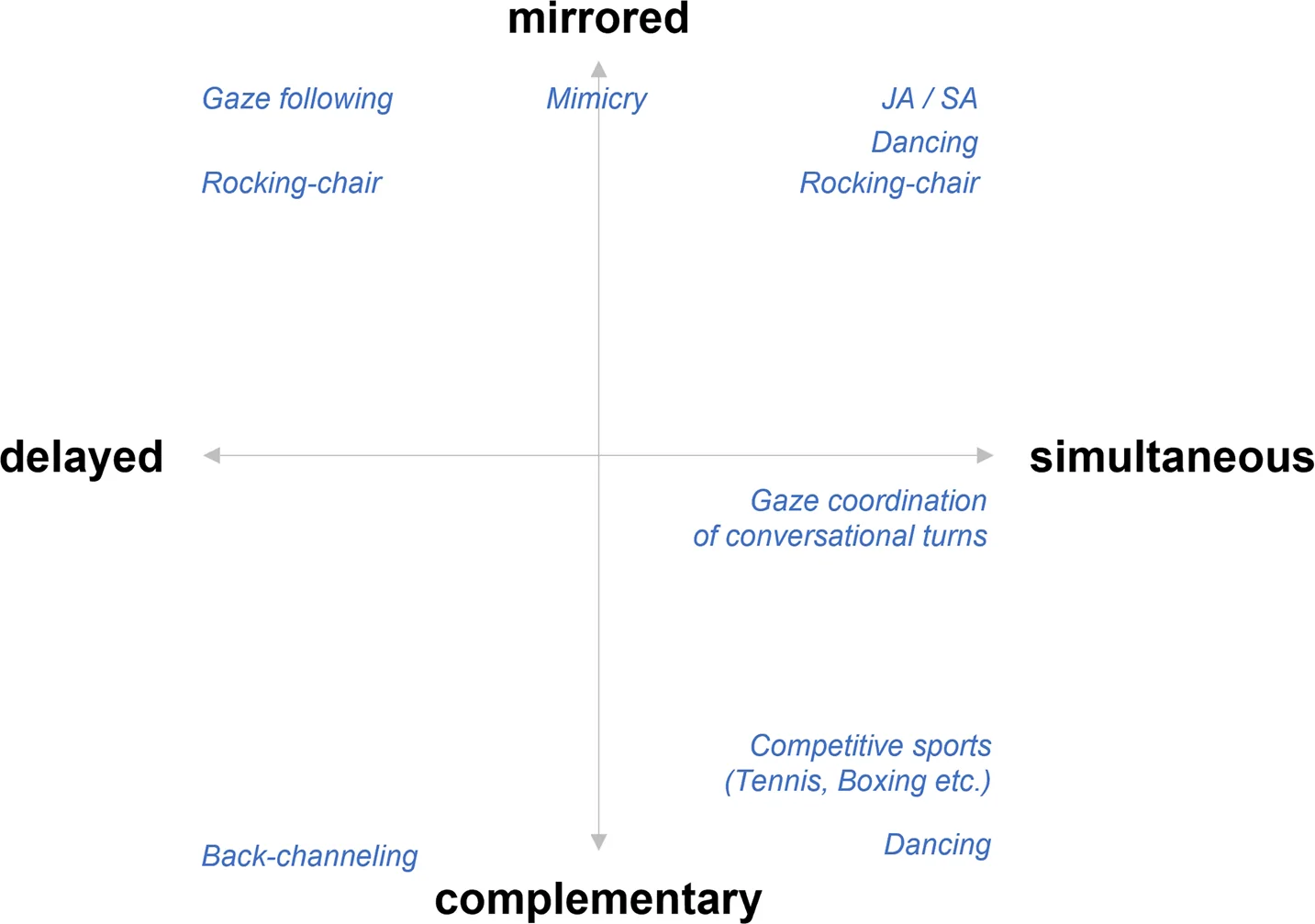
Phenomena of interpersonal synchrony. Types of interactions differ depending on whether interaction partners act simultaneously or with a delay (*x*-axis) and whether the actions are mirrored or complementary (*y*-axis). Note that many of the depicted phenomena themselves might differ to some degree with regard to their simultaneity (*x*-axis) and/or complementarity (*y*-axis) and thus cannot be precisely localized in the diagram. Short descriptions of temporal requirements and expectations of the individual phenomena from left to right, top to bottom: *gaze following*: when trying to lead another person's gaze towards a specific target, gaze following is expected within specific temporal windows [31]; *mimicry*: when mirroring another person´s behaviour (e.g. facial expression), the mirroring person tries to observe and copy the other interlocutor as closely as possible to convey the appearance of similarity [49]; *JA/SA*: joint attention (JA) describes situations in which two interactants focus the same object at the same time, while shared attention (SA) additionally requires mutual awareness of the fact that both persons have the same focus [26,33] which requires additional gaze exchanges [31]; *rocking chair*: both interactants perform the same movement to move their chair, either in simultaneity or in perfectly opposite phases [20]; *dancing*: both interactants move at the same time either with mirrored movements or with complementary movements; *gaze coordination of conversational turns*: speakers can announce the near end of their own utterance and thus the upcoming partners’ turn by looking at the listener or signalling their intention to continue speaking by averting their gaze [43]. Whereas listeners signal their continued attention by looking at the speaker [29]; *competitive sports:* most competitive sports require closely observing the opponent and constantly adjusting one's own behaviour to counter the opponent, resulting in simultaneous but complementary movements by both opponents. *Back-channelling:* in conversations, listeners often periodically signal their understanding by short utterances (‘mhm’, ‘ah’ and ‘ok’) during breaks in the speaker's monologue [50].

Besides the interpersonal coordination, we also need to take into account that the different communicative modalities require the temporal alignment of different communicative channels in the framework of chronemics, for example pointing gestures and gaze behaviour within the person or on a subpersonal level. The integration on a subpersonal level has sometimes also been conceptualized as *intra*personal synchronization as opposed to *inter*personal synchronization [51]. The so-called social entrainment model proposes that the subpersonal formations of temporal patterns or rhythms are aligned in phase and period in order to contribute to the interpersonal communication behaviour [40]. This leads to the assumption that the efficiency and success of communication depend on the coordination of communicative signals on a subpersonal level within critical time windows.

This emphasis on critical time windows applies most directly to face-to-face communication in co-presence of both interlocutors. Mediated and/or asynchronous forms of communication (e.g. phone calls, video-based meetings, text messaging, forums and online lectures) impose different temporal constraints. In such settings, some aspects of multimodal temporal alignment are reduced or even absent, whereas other temporal features of chronemics may become more important (e.g. message pacing, sequencing, pauses, repair opportunities during ongoing conversations and expectations about the interlocutors’ availability). Depending on the preferred communication style (e.g. in autistic persons), asynchronous or reduced-cue formats may lower the demands on rapid (temporally) online integration of cues such as gaze, prosody, gesture and speech [52–54]. At the same time, the reduction or even absence of nonverbal signals may create further ambiguities. Thus, if we talk about atypical communication styles as in autism spectrum disorder (ASD), time-lagged communication can be successful not despite chronemics, but because it follows a different temporal organization that allows a sequential processing and does not require a simultaneous processing.

Finally, the temporal perspective of causality is another important facet of chronemics. Taking intentionality and goal-directedness of persons seriously, communication is extended by finality or teleology, as inspired by theoretical accounts, including ‘critical personalism’ [55], ‘field theory’ [56] and ‘general systems theory’ [57]. Whereas causality refers to reasons of behaviour that lie in the past, teleology refers to goals and purposes of behaviour directed towards the future. Only this complementarity of past-related causes and future-directed goals enables adaptivity to the ever-changing affordances of our environment [55,57]. In this vein, classical field theory by Kurt Lewin proposes a ‘principle of contemporaneity’ according to which behaviour always comprises both time perspectives in a given situation (including the person and their psychological environment) at a given moment (in the sense of a time slice of a certain extension, yet to be defined in its concrete extension). In the case of action planning, we are always integrating causes of what has happened in the past with our wishes and aims to be achieved in the future [58]. We can imagine, for example, a musician playing a melody on his/her instrument. The parts of the piece that have already been played can be traced back to playing the instrument (causality), while the parts of the piece that are still missing refer to goals that will be achieved in the near future (finality) by continuing to play the instrument [59]. Similarly, if person A in a conversation starts smiling, interlocutor B might either assume that A spontaneously remembered a funny episode from their past or that they start smiling in anticipation of something funny they expect B to say. The temporal dynamics of the smile could be expected to differ in those two instances, with a spontaneous broad smile that subsequently fades out in case of the funny memory or an initially slight smile that then broadens with every word by B when in anticipation of a funny story.

## 4. Rapport as a measure of successful communication

Besides content, communicative encounters always comprise a very strong relational component corresponding to the second axiom of communication according to Paul Watzlawick; even unintended signals are usually perceived as meaningful [9]. While we communicate, we experience whether the ongoing communication is successful in the sense of being pleasant or gratifying or how we come along with the interlocutor or how well we are attuned to each other. This experience is also described as ‘rapport’ or ‘connectedness’ or ‘attunement’ with someone else. Rapport necessarily relies on dyadic interactions; it is based on mutual attentiveness, the reciprocal exchange of positivity cues and coordination of nonverbal behaviours [18,60–63]. Accordingly, rapport is the goal and indicator of a successful interplay of interpersonal and intrapersonal synchronization and alignment of multimodal communication signals. Of highest relevance to experiencing success of communication is the ‘gratification that an exchange of messages provides for the participants […] To be understood is a pleasure; to reach an agreement is expedient and pleasant; to be understood and to reach agreements is deeply gratifying’ [64, p. 36]. Temporal alignment often happens automatically and seemingly effortlessly but can depend on dispositional empathy of interactants [49]. Vice versa, mutual temporal alignment of nonverbal communication increases the experience of rapport [18,21,61,65,66], strengthens mutual affiliation [67], enhances mentalizing [68] and empathy [69,70] and supports the success of joint action [71,72].

Besides indicating a pleasant atmosphere, rapport also has very concrete correlates: patients who experience strong rapport with the therapists report higher satisfaction with their treatment [73] and show a higher degree of commitment and compliance during therapy [74] thereby supporting the constitution of a therapeutic alliance. The subjective experience of rapport can correlate with motor and postural mimicry and the temporal entrainment or interpersonal synchrony of communication partners [21,75,76]. Interpersonal synchrony can demonstrate prosocial effects [66,67,77], but it can also impede self-regulation processes [78]. Conceptualizing an adaptive interpersonal system, it has been postulated that adaptivity requires two different capacities of a person that exist simultaneously, namely the tendency to synchronize with others in the interplay with the tendency to move out of synchrony and act independently [79]. In other words, synchrony as a particular type of interpersonal coordination may not always be beneficial for the interaction partners.

## 5. Variance of communication: successful and disturbed communication

Communication research in its majority has been focusing on communication that is experienced as goal-directed, pleasant or ‘successful’ [80,81]. Interestingly, the variability with which we achieve successful communication is virtually infinite owing to the variety of languages, cultures and settings and owing to individual skills, styles and repertoires of the communication partners. As a consequence, the conveyed messages may differ substantially. For example, the social norms that determine whether a contact request from another person should be responded to immediately or only after a delay may depend on the nonverbal channels used (mutual gaze and shaking hands). To allow for both a high degree of creativity in our communicative expressions, on the one hand, and the capacity of repair or the ability to resolve irritations and misunderstandings, on the other hand, our communication abilities must be robust but also flexible at the same time. The flexibility of our communication makes it susceptible to breakdowns and might require it to apply repair mechanisms. This is relevant for ‘running repairs’, namely repair mechanisms that try to deal with semantic misunderstandings during running conversations [82]. Repair also plays a key role in the acquisition of language [83]. As a consequence of our ability to repair and the resulting flexibility of signal exchange, communicative encounters vary substantially.

However, the adaptivity that allows adequate responses to the partner can get lost. Accordingly, it has been proposed that the variance of human experiences and behaviour reflected in psychopathological conditions can be reconstructed as disturbances of communication [8,64,84–87], in contrast to classical psychopathological accounts according to which only the individual is affected to whom the mental disorder is attributed [88]. Psychopathological symptoms (perceptions, belief systems, emotional experiences and communicative behaviour) all refer to a social community as a background against which the subjective experience of the person suffering from a mental disorder stands in contrast [85]. As a consequence, the communicative account should be the central and fundamental concept in psychopathology [8,64,84].

Especially, communication of persons under psychopathological conditions is in its complexity only poorly understood. Already decades ago, it was postulated that communication plays a key role in the understanding of psychopathological conditions: ‘Psychopathology is defined in terms of disturbances of communication’ [8, p. 79], accordingly the ‘only thing that the psychiatrist really can rely upon is the state of communication as it is observed at a given moment, within a given context, and involving specific people’ [8, p. 91]. This implies that the scientific focus on communication has to include variances of communicative behaviour. It has been suggested to conceptualize variances between the poles of successful communication and disturbed communication. Whereas successful communication is supposed to be characterized by a ‘mutual fit of overall patterns and constituent parts, … efficiency of operation, … flexibility of network and procedures’ resulting in ‘gratification for participants’, disturbed communication, in contrast, refers to the ‘the quantitative deviation and the inappropriate pattern’ resulting in ‘frustration’ [64, p. 49]. In short, disturbed communication, according to this view, is nothing else but the ‘improper timing of messages’ [64, p. 49].

Although social cognition has become a central theme in research on psychopathological conditions with respect to ASD [87,89] or schizophrenia [85,90], the majority of studies focused on ‘offline’ social cognition of detached and isolated individuals but have not studied sufficiently ‘online’ ongoing communication involving a second-person perspective [91]. Persons with schizophrenia exhibit communicative behaviour and traits that often overlap with ASD, including motor coordination deficits, reduced interpersonal synchrony and shared mentalizing deficits [92–97]. At the same time, the prevalence of depression is high in ASD [98], and symptoms of major depressive disorders also affect nonverbal behaviour and interpersonal alignment [99–105]. It is an unresolved research agenda to explore which of the phenomena of disturbed communication are shared between different conditions and which are specific to the different diagnostic groups, especially under differential temporal aspects: to what extent does the temporality of cues differ in diagnostic groups and contribute to the experience of ‘disturbed’ communication?

## 6. Autism spectrum disorder as a case of disturbed communication

### (a) Individual mindblindness or dyadic disturbance

Traditionally, patients with ASD have been treated as persons suffering from ‘mindblindness’ [106] with lifelong and stable deficits in nonverbal communication and social interaction, while verbal and general learning and memory abilities develop independently and are often fully preserved. More recently, classical ‘theory of mind’ accounts have been discussed critically in two review publications [107,108]. The general claim that autistic people are uniquely impaired in theory of mind tasks cannot be generally confirmed, and theory of mind deficits do not relate to social interaction and empathy [107]. A very detailed analysis of the classical theory of mind revealed that the propensity to infer mental states and their accuracy are key to understanding the heterogeneous results of theory of mind research in autism [108]. The ‘mind-space’ concept [109] suggests that different persons will experience the same situation differently. According to this concept, a multidimensional space has to be postulated which would allow the differential representations of traits of persons [109]. A person who tries to infer the mental states of another person would then need to capture the covariation of these traits and could potentially infer the mental state, making use of ‘a set of experience dependent learned relationships’ [108, p. 1019]. This detailed account has already stimulated a new interview-like task [110]. Although this approach develops the theory of mind approach substantially further and can potentially stimulate clinical interview techniques in the interaction with patients, this approach is still much more focused on social perception in a theory of mind context but does not take a full-fledged dyadic perspective.

Behavioural and neural research findings show consistently atypical processing of socially relevant information and a decreased salience of social cues in ASD, including mentalizing [111–114], detection of emotions [115], social motion evaluation [116], gaze perception [117], person perception [118], perception and production of prosody or intonation [50,119,120] and the detection of interactive intentions [121–123]. On a neurobiological level, this was confirmed by the reduced neural activation of the mentalizing system encompassing the medial prefrontal cortex, posterior cingulate cortex and temporoparietal junction in persons with ASD in gaze perception tasks [124,125] and in an animacy perception task [126]. Besides these very focused studies, there are also proposals that are exploring the field from a meta-analytical perspective of ‘relational neuroscience’ [127]. However, an extended study on autistic persons (*n* = 205) in contrast to control persons (*n* = 189) did not show any difference in neural activation of the ‘social brain’ (dorsomedial prefrontal cortex, posterior cingulate cortex and temporoparietal junction) making use of an animated shape task [128], possibly owing to the large heterogeneity of this multicentre study.

Recent work, however, has substantially expanded the study of interaction deficits in ASD by including different types of dyads in their sample, namely homogeneous autistic dyads, homogeneous non-autistic dyads and heterogeneous dyads with one autistic and one non-autistic person across naturalistic, experimental and dyadic settings [129–134]. Interactional outcomes cannot be fully explained by individual autistic deficits alone but depend on dyadic composition, task demands, communicative modality and the specific measure under investigation. Supporting the key role of the dyad, it was shown that autistic and non-autistic adolescents show stronger same-neurotype peer preferences over time, and same-neurotype interactions appear more likely to be relational, reciprocal and characterized by sharing thoughts or experiences than cross-neurotype interactions [131]. Experimental work further suggests that autistic adults may be able to generate and modify intelligible communicative behaviours, while experiencing difficulties when successful interaction requires dynamic alignment with a partner's recent communicative history [129]. Studies of dyadic interaction also show that rapport, gaze behaviour, smiling synchrony, utterance length, and other behavioural or kinematic indices may vary across the two types of homogeneous and heterogeneous dyads [132,133]. Taken together, these findings strengthen the need to systematically compare all three dyad types directly rather than attributing interactional difficulties to one individual in isolation.

### (b) Double empathy

In contrast to the view of deficits in social cognition in individuals with ASD, the so-called double empathy problem was proposed [135,136]. The basic idea is that not only persons with ASD lack empathy but also their non-autistic counterparts in dyadic interactions. Accordingly, the double empathy problem is defined as a ‘disjuncture in reciprocity between two differently disposed social actors which becomes more marked the wider the disjuncture in dispositional perceptions of the lifeworld’ [135, p. 883]. In simple terms, it ‘refers to a breakdown in mutual understanding (that can happen between any two people) and hence a problem for both parties to contend with, it is more likely to occur when people of very differing dispositions attempt to interact’ [136, p. 1901]. According to this theory, putative social deficits of persons with ASD are, in fact, mutual misunderstandings that lead to disturbed communication as a consequence.

An interesting study showed adequate communication behaviour in the interaction between autistic persons comparable to communications between non-autistic persons during an information transfer task. Information transfer was worse in mixed dyads between autistic and non-autistic persons; interestingly, rapport ratings were also lower in mixed dyads. Findings were interpreted as confirmation of the double empathy theory insofar as autistic persons appear to have the skills to share information among themselves, whereas problems arise only in mixed dyads [137,138]. Indeed, persons without ASD show difficulties in interpreting facial expressions, mental states or social intentions of autistic persons [139–141], but these difficulties could possibly also be attributed to the weaker nonverbal signals that persons with ASD express which is also detected by autistic persons. It seems that persons with ASD also express distinctive communication patterns among themselves such as atypical prosody or reduced eye contact [142]. It could be shown that persons with ASD tend to choose other autistic persons as potential interaction partners even if they appear more awkward, less attractive and less socially warm [143]. However, in contradiction to the expectations of the double empathy theory, no differences were observable depending on the diagnosis of the partner, that is whether that partner of a person with ASD was an autistic or a non-autistic partner, when comparing synchrony between partners in terms of motion energy [130]. An interesting study explored the nonverbal communication behaviour in all three types of dyads, namely homogeneous autistic, homogeneous non-autistic and heterogeneous or mixed dyads. Although the study was designed and interpreted in the framework of double empathy problem, it did not show any difference between homogeneous autistic and mixed dyads: both groups of dyads showed reduced backchannel rates and reduced mutual gaze compared to homogeneous non-autistic dyads [144].

### (c) Dialectic misattunement hypothesis

A proposal closely related to ‘double empathy’ is the dialectical misattunement theory [86], which conceptualizes autistic communication difficulties not merely as individual deficits but as dynamic mismatches between interacting persons. This perspective is also highly compatible with the general intuition conveyed here that the dyad needs to be taken into account if successful and disturbed forms of communication are to be explored scientifically. According to the dialectic misattunement hypothesis, mental disorders, including ASD, are not viewed as static conditions that are only driven by one causal factor but should be rather understood as the product of a complex interplay of multiple factors. It is assumed that these factors interact with each other in a ‘circular causality’ and are to be identified on different levels of description that encompass not only biological and cognitive-behavioural but potentially also socio-cultural levels. Further, the dialectic misattunement includes the development over time and can potentially cover evolutionary, cultural, social, personal and subpersonal biological time scales [86]. With respect to ASD, this account understands the condition as ‘cumulative misattunement between persons, which can be thought of as disturbances in the dynamic and reciprocal unfolding of an interaction across multiple time scales, resulting in increasingly divergent prediction and (inter-)action styles’ [86, p. 357]. The interdependence of individual and collective levels of description is emphasized. Accordingly, interactions within homogeneous dyads of two autistic or two non-autistic persons are expected to appear smoother compared to heterogeneous dyads between one autistic and one non-autistic person. This implies that the dialectic misattunement account makes similar assumptions compared to the double empathy theory. It is further assumed that already during early ontogenetic development such a misattunement could already lead to reduced acquisition of social skills. In a dialectic fashion, two processes, namely a weaker coupling between interaction partners on a social level and different communication styles at the individual level, come together and result in the misattunement of both partners. Consequently, ‘attunement’ is considered essential for successful communication.

These different factors are to be studied in the framework of predictive processing and the active inference account assuming that the brain calculates and provides probabilities about events in the environment or about states of the self on the basis of previous experiences and newly sensed information. On this basis, ASD is reinterpreted as a different prediction style resulting in different communication behaviour. Misalignments in communication might result. Taken together, the dialectic misattunement account shifts the focus of scientific attention towards ‘a multilevel observation of dyads and groups of interactors’ [86, p. 369].

### (d) Double invisibility

We propose an alternative perspective of ‘double invisibility’, which becomes apparent in the interaction between an autistic person and a non-autistic person. The key factor here is the discrepancy between the partners’ differing implicit mutual expectations regarding their intuitive and automatic communication. It brings together the fundamental deficiency of producing and perceiving nonverbal communication signals in an automatic and intuitive fashion in ASD and the concept of the dyad as the fundamental unit of the analysis. This deficiency can be interpreted as a variant of the social self in ASD. The important aspect to be emphasized here is the automatic and intuitive processing of nonverbal signals that is particularly affected in ASD [145,146]. This distinction between propositional formats (verbal descriptions) and non-propositional formats (nonverbal signals) is essential for the understanding of the nature of ASD. Nonverbal communication does not have an explicit semantic code and is complex owing to the co-occurrence of different communicative cues [147]. It is important to note that propositional or explicit processes do not necessarily overwrite non-propositional processes; in other words, non-propositional processes are still highly relevant even if they contrast with propositional processes [148]. This is in accordance with classical communication theorists claiming that it is the relational aspect that shapes the dyad and allows it to convey messages within this space of successful communication [9]. Verbally conveyed information that refers to an explicit semantic code is usually well recognized, adequately processed and even used as a compensation strategy by persons with ASD. However, since the processing of nonverbal communication is a predominantly intuitive or automatic process outside our awareness [149], these nonverbally conveyed communicative signals are usually not accessible to conscious insight or are difficult to access and definitely not in their full complexity and simultaneity. Non-autistic individuals constantly use nonverbally conveyed social signals without being permanently aware of their use of them [150] and—owing to their inherent complexity—cannot be fully aware of them at any time [11]. People with ASD, on the other hand, have significantly more difficulty or often no access at all to this intuitive means of communication [145,146]. Accordingly, in gaze interactions with a virtual partner, participants with and without ASD did not differ in the allocation of visual attention towards socially relevant regions and engaged equally frequently in joint attention with their partner [122]. Nonetheless, it seems that persons with ASD were less likely to recognize that occurrences of joint attention were the result of a joint effort of interpersonal synchronization instead being of incidental.

Metaphorically speaking, one can also refer to this observation as the ‘double invisibility’ that emerges during the interaction between an autistic and a non-autistic person. Besides the trivial observation that a certain invisibility is common to all mental disorders as they concern predominantly the inner experience of the affected person—in contrast to somatic illnesses—there are two more subtle but specific facets of invisibility that shape the interaction between a non-autistic and an autistic person. Non-autistic people intuitively expect rich, multimodal communication that includes nonverbal cues; they generate, process and expect nonverbal cues without being aware of it (first invisibility). By contrast, autistic people generate, process and expect nonverbal cues either to a significantly lesser extent or not at all (second invisibility). Neither partner is aware of their mutually incompatible expectations and abilities regarding intuitive nonverbal communication, so these expectations remain invisible. Thus, the two facets of invisibility arise from the automatic and unconscious assumption in everyday life that all people have the same intuitive automatic understanding of social signals as oneself, while individuals who may not be able to communicate as intuitively or follow different communicative styles only with great difficulty—as is the case with ASD—are overlooked. This would be especially apparent in interactions with persons with autism who rely especially little on nonverbal cues in their communication. These two invisibilities are therefore a specific feature of the interaction with persons with ASD.

Interestingly, it appears that in the case of speech behaviour, specifically turn-taking behaviour, persons with ASD are indeed able to adapt to the turn-taking behaviour of their non-autistic partner during the course of a conversation. However, this attunement was achieved only after a certain period of time, suggesting that persons with ASD, given the opportunity and enough time, recognize their different speech behaviour and try to adapt [151]. This finding raises the interesting research question of whether non-autistic interlocutors may likewise adapt to autistic communication styles when given sufficient time and awareness of relevant differences. Except in non-symmetrical interactions between, for example, carers or professional clinicians and autistic children or adolescents [36,152–154], only little empirical evidence exists for non-autistic persons adapting to their autistic interlocutors, for example spontaneous imitation of speech rhythms of non-autistic individuals if they interact with autistic and non-autistic interlocutors [155].

So the double invisibility metaphor takes into account—as the double empathy theory and the dialectic misattunement hypothesis—that the misunderstandings in interactions between persons with and without ASD are involving both interlocutors based on the conceptualization that the dyad is the fundamental unit of the analysis. However, it is not primarily owing to a lack of empathy of any of the two partners, but rather a matter of the invisibility of the complexities of nonverbal communication owing to the intuitive, automatic and seemingly effortless processing of nonverbal signals. The double invisibility metaphor is also, in principle, in line with the proposal of more detail-focused and less holistic cognitive style as postulated in the ‘weak central coherence’ proposal of autism [156].

## 7. Future research directions

On the basis of the presented research on subpersonal and interpersonal integration of multimodal communication cues within the social self, the following aspects for possible future directions need to be considered. The research clearly shows that the basic unit to be explored is the dyad when it comes to the study of interaction and communication. Although studies on person perception or impression formation have been very instructive, it is obvious that they cannot resemble the high dimensional and processual complexity that is required for the ‘online’ processing of a multitude of different communicative cues during face-to-face conversations. During listening and registration of the different cues, the next utterance has to be prepared in order to respond in a fluent manner, usually only after a few hundred milliseconds. This complexity has to be taken into account adequately, and it can only be studied in ongoing conversations in dyads. It has been suggested that the interplay between subpersonal and interpersonal processes contributes to the experience of ‘attunement’ or ‘connectedness’ with the other measurable as rapport experience. Even recent studies on dyads still neglect the question of how these observable traits, in turn, influence the ongoing communicative dyad itself in a recursive manner [86,87]. Acquisition of such interactional data would require set-ups with high ecological validity. In addition to the set-up, it is challenging to elicit natural, interactional, dyadic communicative situations with conversations that should have a genuine speaking intent.

After focusing on dyads as the fundamental unit of analysis, it is very important to study different types of dyads as some studies have already shown. Studies should ideally include both types of homogeneous dyads between two non-autistic and two autistic persons as well as heterogeneous dyads between one autistic and one non-autistic person [130,157]. This is the only way to disentangle the effects of personal preferences in communication styles in persons with ASD from misunderstandings because of different expectations and requirements in dyads. For more than a decade, this issue has been discussed as the double empathy problem, assuming that it is not only one of the two partners who lacks empathy but empathy is lacking in both interlocutors. The ‘double invisibility’ metaphor instead accounts for both individual differences in persons with ASD in the processing of intuitive, automatic nonverbal communication cues and, at the same time, the incompatibility between both partners because of implicit competences and expectations. Whether this is a more helpful view can only be explored in systematic studies of all three different types of dyads in a kind of series of dilutions. Accordingly, it should be emphasized whether the focus of a study is on propositional versus non-propositional formats.

Methodologically, the multimodal stream of communication requires new measurement set-ups that allow the registration of and integration of multiple cues and data streams. We ourselves have started to work on the interplay between gaze and speech behaviour [158,159]. Gaze and speech are especially interesting with respect to turn-taking behaviour [43]. Technically, integrating multiple cues into one data format with a shared time stamp is not trivial, but this is highly interesting to explore the interplay and mutual influences of nonverbal cues.

Another nearly completely missing field of research is the interplay between subpersonal and interpersonal information or the interplay between individual self and social or interpersonal self. In a study on time-coordinated actions in a social and non-social task, it could be shown that the synchronization behaviour was different between persons with and without ASD insofar as associations between both tasks were visible only in persons without ASD, whereas these cross-domain associations were largely reduced in the autistic population [160]. Possibly related is the observation that the subpersonal integration of gaze and deictic gestures is less coherent in persons with ASD [161]. In a follow-up study, using exactly these temporal patterns projected onto neutral virtual characters in a communication task, we could show that the decoding times were longer when virtual characters expressed an autistic phenotype, and that this effect was even more pronounced in observers with ASD [160]. This suggests that subpersonal differences of the temporal alignment or the ‘chronemics’ of multimodal communication cues affect individuals with and without ASD differently and can lead to subtle changes on the interpersonal level during encounters of persons with and without ASD. In more complex interactions where participants were able to communicate verbally in addition to gaze, persons with ASD seemingly also differed from non-ASD participants in the allocation of attention, focusing less on the eye region and more on the mouth [162]. Possibly, participants with ASD had difficulties integrating the two nonverbal channels, gaze and speech, and instead focused mostly on speech alone. The unidirectional focus on one communication channel consequently reduces drastically the complexity of interactional chronemics and requirements towards inter- and intrapersonal synchronization. This interplay between social and non-social tasks focusing on the integration of sub- and interpersonal processes or individual and social self is a nearly entirely neglected field in research.

## 8. Conclusions

These considerations show that the social self is not a static property of a detached individual, but it is continuously constructed and expressed through the real-time, multimodal coordination between interlocutors that we have described above. Viewed this way, ‘double invisibility’ describes not only a communication difficulty but also a disruption of the constitution of the social self in persons with ASD. Because autistic and non-autistic partners generate, process and expect nonverbal cues differently without either partner being mutually aware of the mismatch, the moment-by-moment co-construction of the social self between them can become attenuated or skewed, even though each partner's individual self remains intact. Understood in this way, the diversity of the social self in ASD reflects not a deficit located within one individual but a property of the dyad's capacity for real-time attunement.

Besides research, the above proposed considerations have a number of implications. If it were possible to obtain multimodal communication streams with simple tools, combining automatic and continuous recordings of different multimodal signals, this would have potentially a great impact on the diagnosis of ASD, alongside competent clinical interviews and structured examinations (Autism Diagnostic Observation Schedule-2) [163]. However, a quantification of temporally atypical coordination of communication signals in ASD is not only an important explanatory approach that potentially informs diagnosis as well as intervention programmes but could also be used for training interventions of successful nonverbal communication. Finally, the concept of ‘double invisibility’ has also potentially important therapeutic implications during which persons with ASD could be taught to develop their perception of nonverbal cues. A very first step in providing appropriate care must be to draw attention to the particularities of double invisibility, both among professional psychotherapists and the general public. When planning treatment, a second step should be to focus on the specific needs of people with ASD in psychotherapy in the context of developing needs-oriented group psychotherapy for adults with ASD [164].

## Data Availability

This article has no additional data.

## Theme

One contribution of 17 to a discussion meeting issue ‘From being to identity: analysis and synthesis of the self’.

## Glossary

### Core concepts

Individual self: (private self, self as subject, ‘I’) refers to self-reflective, introspective or contemplative states, in which one reflects on oneself and one's own inner states and mental condition [1–4]. The related cognitive capacity is self-other differentiation [5].
Social self: (interpersonal self, self as object, ‘Me’) refers to interactions and communication with others, in which one also has to take into account the inner states of others and how oneself is perceived by others [1–4]. The related cognitive capacity is self-other exchange [5]. In a cultural context, an independent self that stresses the individual's independence from others (individualism) is distinguished from an interdependent self that stresses the fundamental relatedness to others (collectivism) [5,6].
Communication: (lat. communicare: to do or put something together) refers to the dynamic exchange or sharing of information or knowledge between two communication partners conveyed by multimodal meaningful communicative messages and based on our mentalizing abilities. Communication coordinates interaction, including discourse and dialogue, manages attention, and establishes common ground [8,9,150].
Interaction: (lat. inter, agere: acting between) comprises any mutual reciprocal action or influence between persons or entities and focuses on the relationship between them. Interaction can involve communication.
Interpersonal coordination: (lat. organize together) refers to the general process by which individuals adjust their behaviour relative to one another during social interaction. This adjustment can result in simultaneous, alternating, complementary or sequential patterns of behaviour, such as turn-taking in conversation or adapting one's speech rate to a conversational partner [38,78].
Interpersonal synchrony: can emerge, often spontaneously, as a measurable state or feature (on the basis of the process of synchronization) and is characterized by temporally aligned behaviour (e.g. matching speech rates, similar prosody and simultaneous movements). It does not take into account the experiential level [39].
Social entrainment: corresponds to the process of interpersonal synchronization resulting in interpersonal synchrony as a state of temporally aligned behaviour [40].
Intrapersonal synchrony: describes the measurable feature of temporal alignment of different communication channels (e.g. intonation, gestures and gaze behaviour) within a person (on the basis of the process of synchronization during intrapersonal coordination). It does not take into account the experiential level [49,160,161].
Rapport: represents the gratifying and positive experience of attunement and getting along well with a communication partner and corresponds to the experience of ‘successful communication’. Rapport is an emergent property of the dyad as the basic unit of the analysis and relies on the dynamic coordination and exchange of behavioural, affective and cognitive signals within dyads [18,58–61].
Chronemics: refer to the study of concepts and processes of human temporality covering either objective time (physical clock time) or subjective time (experienced structure of time and passage of time) as they play a role for human communication. The temporal structure of communication includes a past (related to causality), the present and the future (related to finality or goal-directedness). Different modalities operate on different time scales (e.g. gaze: milliseconds; body posture: seconds) [46–48].

### Theoretical accounts

Double empathy problem: assumes that in heterogeneous dyads not only one partner (e.g. person with autism) but both partners (persons without autism) lack empathy for their interlocutor from the other group, resulting in a breakdown of mutual understanding [135,136].
Dialectic misattunement: proposes that psychiatric conditions are to be understood as the result of a dialectic process integrating individual and collective factors in a circular causality among different levels of description (e.g. biological, cognitive-behavioural and socio-cultural) with different functions within a given level (e.g. action and perception within the cognitive-behavioural level), unfolding in time (e.g. evolutionary, cultural, social, individual-psychological and subindividual-biological developing scales). Disturbances of these multiple dialectic processes can lead to misattunements between persons and can result in divergent prediction and (inter-)action styles. It is based on the account of predictive processing and active inference [85].
Double invisibility: focuses on the processing of nonverbal communication, which is usually automatic, intuitive, unconscious and effortless (‘invisible’). Non-autistic people intuitively expect rich, multimodal communication, including nonverbal cues; they generate, process and expect nonverbal cues without being aware of it (first invisibility). By contrast, autistic people either generate, process and expect nonverbal cues only to a significantly lesser extent or not at all (second invisibility). This results in a mismatch of communicative styles and misunderstandings.
